# Bioluminescent Imaging Reveals Divergent Viral Pathogenesis in Two Strains of Stat1-Deficient Mice, and in αßγ Interferon Receptor-Deficient Mice

**DOI:** 10.1371/journal.pone.0024018

**Published:** 2011-09-07

**Authors:** Tracy Jo Pasieka, Lynne Collins, Megan A. O'Connor, Yufei Chen, Zachary M. Parker, Brent L. Berwin, David R. Piwnica-Worms, David A. Leib

**Affiliations:** 1 Department of Ophthalmology and Visual Sciences, Washington University School of Medicine, St Louis, Missouri, United States of America; 2 BRIGHT Institute, Molecular Imaging Center, Mallinckrodt Institute of Radiology, Washington University School of Medicine, St Louis, Missouri, United States of America; 3 Department of Microbiology and Immunology, Dartmouth Medical School, Lebanon, New Hampshire, United States of America; Southern Illinois University School of Medicine, United States of America

## Abstract

Pivotal components of the IFN response to virus infection include the IFN receptors (IFNR), and the downstream factor **s**ignal **t**ransducer and **a**ctivator of **t**ranscription 1 (Stat1). Mice deficient for Stat1 and IFNR (Stat1^−/−^ and IFNαßγR^−/−^ mice) lack responsiveness to IFN and exhibit high sensitivity to various pathogens. Here we examined herpes simplex virus type 1 (HSV-1) pathogenesis in Stat1^−/−^ mice and in IFNαßγR^−/−^ mice following corneal infection and bioluminescent imaging. Two divergent and paradoxical patterns of infection were observed. Mice with an N-terminal deletion in Stat1 (129Stat1^−/−^ (N-term)) had transient infection of the liver and spleen, but succumbed to encephalitis by day 10 post-infection. In stark contrast, infection of IFNαßγR^−/−^ mice was rapidly fatal, with associated viremia and fulminant infection of the liver and spleen, with infected infiltrating cells being primarily of the monocyte/macrophage lineage. To resolve the surprising difference between Stat1^−/−^ and IFNαßγR^−/−^ mice, we infected an additional Stat1^−/−^ strain deleted in the DNA-binding domain (129Stat1^−/−^ (DBD)). These 129Stat1^−/−^ (DBD) mice recapitulated the lethal pattern of liver and spleen infection seen following infection of IFNαßγR^−/−^ mice. This lethal pattern was also observed when 129Stat1^−/−^ (N-term) mice were infected and treated with a Type I IFN-blocking antibody, and immune cells derived from 129Stat1^−/−^ (N-term) mice were shown to be responsive to Type I IFN. These data therefore show significant differences in viral pathogenesis between two commonly-used Stat1^−/−^ mouse strains. The data are consistent with the hypothesis that Stat1^−/−^ (N-term) mice have residual Type I IFN receptor-dependent IFN responses. Complete loss of IFN signaling pathways allows viremia and rapid viral spread with a fatal infection of the liver. This study underscores the importance of careful comparisons between knockout mouse strains in viral pathogenesis, and may also be relevant to the causation of HSV hepatitis in humans, a rare but frequently fatal infection.

## Introduction

Herpes simplex virus type (HSV) is a ubiquitous human pathogen capable of causing significant morbidity in immunocompetent patients. Primary and recurrent infections most often cause orofacial lesions, genital lesions, or in the case of ocular infection, herpetic stromal keratitis. Disease in immunocompetent individuals, however, is usually self-limiting. Patterns of disease in immune-compromised patients are often more severe, and in particular, neonates may suffer disseminated infections following HSV infection, with involvement of the skin, eye, mouth, central nervous system, liver, lung, and adrenal glands [1]. This widespread infection is attributed to the immature T-cell and IFN responses in neonates as compared to adults [2], [3]. Consistent with this, adults with impaired IFN Type I and Type II responses, due to either a deficiency in the signal transduction and transcription factor 1 (Stat1), Toll-like receptor 3 (TLR3), or UNC-93B (an endoplasmic reticulum protein important for TLR signaling), show increased susceptibility to HSV and other viral infections [4], [5], [6], [7]. In addition, immune-suppressed and immune-compromised patients show increased susceptibility to HSV hepatitis and can develop acute liver failure [8], [9], [10].

Stat proteins are transcription factors that regulate immune and growth processes [11]. In particular, Stat1 is a critical component in both Type I and Type II IFN receptor signaling. IFN binding to its cognate receptor activates kinases that phosphorylate Stat1. Following Type I IFN receptor signaling with IFNα/ß, a heterotrimeric complex consisting of pStat1/pStat2/ISGF3 assembles and translocates to the nucleus, wherein it mediates the expression of genes containing IFN-stimulated response elements (ISREs). In Type II IFN signaling (IFNγ), pStat1 forms homodimers that mediate expression from genes containing gamma-activated sequence (GAS) motifs. Genes downstream of the ISRE and GAS elements are crucial to controlling viral infection and initiating the adaptive immune response. Mice and cells lacking these components have helped define these pathways. Two mouse lines have been constructed whose Stat1 gene is lacking either the N-terminal domain (termed here Stat1^−/−^(N-term)) [12] or the DNA binding domain (termed here Stat1^−/−^(DBD)) [13]. IFNR^−/−^ mice lacking Type I and/or Type II IFNR have been used to examine the separate and combined contributions of these receptors [14], [15], [16]. Herein, we use both Stat1^−/−^ and IFNRαßγR^−/−^ mice to study how each component in the IFN signaling pathway contributes to the control of HSV infection.

HSV-1 has several countermeasures to the IFN response, including the viral proteins ICP34.5, US11, ICP0, and vhs [17]. In general HSV is resistant to the effects of IFN, but recombinant viruses lacking any of these proteins show varying degrees of increased sensitivity to host IFN. This study focuses on two mouse strains in the 129 background, 129Stat1^−/−^(N-term) [12] and IFNαßγR^−/−^ (also termed AG129) [15], [16] mice, both of which have been demonstrated to have increased susceptibility to various pathogens, including Listeria monocytogenes, vesicular stomatitis virus, dengue virus, Sindbis virus, mouse norovirus, and mouse cytomegalovirus [12], [18], [19], [20], [21], [22], [23]. The susceptibility patterns, however, vary between these mice, and between pathogens. Stat1^−/−^ and IFNαßγR^−/−^ mice both show increased susceptibility to HSV infection and differences in HSV pathogenesis [24], [25], [26], [27], [28], [29], [30]. Such differences have previously been ascribed to either Stat1 independent signaling of IFNs, [23] or to other non IFN-related effects of deletion of Stat1 [29]. Recent studies have also demonstrated that Stat2 is a major mediator of Stat1-independent host defense to dengue virus [31].

The goal of the present study was to examine further the respective roles of Stat1 and IFN receptors in the control of HSV-1 infection. In addition, we wished to compare two different Stat1^−/−^ mice, namely the mice constructed by Meraz et al. (referred to herein as 129Stat1^−/−^(N-term)) [12], and those made by Durbin et al. (referred to herein as B6Stat1^−/−^(DBD) and 129Stat1^−/−^(DBD))[13], [32]. HSV-1 displayed markedly divergent pathogenesis in 129Stat1^−/−^(N-term) and AG129 mice. Strain 129Stat1^−/−^(N-term) mice cleared a disseminated visceral infection, yet succumbed with high levels of central nervous system (CNS) infection. In contrast, infection of the IFNαßγR^−/−^ mice resulted in a frank viremia that started in the draining lymph nodes and which progressed to a rapidly fatal infection that coincided with an overwhelming infection of the liver and spleen. Furthermore, the ability of the 129Stat1^−/−^(N-term) mice to control the visceral infection was dependent on signaling via the Type I IFN receptor. These results highlight a clear role for the IFN receptors in controlling the extent and tropism of viral replication. Furthermore, the data show that the two Stat1 knockout mouse strains tested are significantly different in their susceptibility to HSV-1, with the mice lacking the N-terminus of Stat1 [12] being significantly more resistant to HSV-1 infection than those lacking the DNA-binding domain [13], [32]. These data demonstrate these two mouse strains cannot be considered interchangeable for virus infection and pathogenesis, and data derived from each of these strains should be compared and interpreted with caution.

## Results

### HSV lethality and disease in control, 129Stat1^−/−^(N-term), and AG129 mice

**Figure 1:**
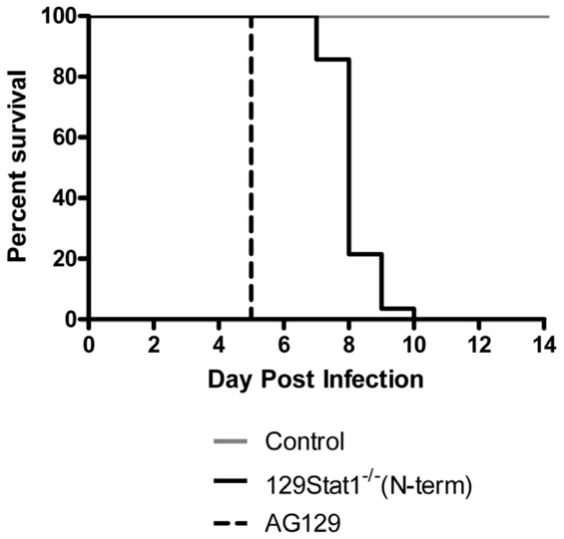
Survival of mice lacking intact IFN signaling. Kaplan-Meier survival plot of mice infected via the cornea with 2×10^6^ pfu/eye of HSV-1 KOS in control (gray line), 129Stat1^−/−^(N-term) (solid black line), or AG129 (dashed black line). The plot represents at least 20 mice per model, derived from at least 3 independent experiments.

In separate studies, strain 129Stat1^−/−^(N-term) and AG129 mice have exhibited a high degree of susceptibility to corneal inoculation of HSV-1 [25], [26]. As previously reported, all infected control mice survived HSV-1 infection with minimal, if any, ocular disease (Fig. 1 and data not shown). Strain 129Stat1^−/−^(N-term) mice died between 7 and 10 dpi with substantial external disease, behavioral signs of CNS infection, and weight loss (Fig. 1, data not shown and [26]). In contrast, HSV-1 virus-infected AG129 mice showed mild ocular disease up to 3 dpi, but this was followed by weight loss, development of a quiet demeanor with little movement or response to stimulation, and remarkably synchronous death on 5 dpi without apparent external disease. Similar timelines were reported for this model system by Halford et al. [30]. These results highlight two important aspects of these infection models. First, as observed for other pathogens 129Stat1^−/−^(N-term) and AG129 mice are not equivalent in their ability to control an HSV-1 infection [12], [18], [19], [20], [21], [23], [30], [33]. Second, and not previously studied, clinical signs prior to death, and timing of death differed substantially between the two IFN response-deficient mouse models, prompting us to examine viral spread and to look more closely at the patterns of pathogenesis and lethality.

### Shift in tissue tropism visualized by *in vivo* bioluminescence imaging

**Figure 2:**
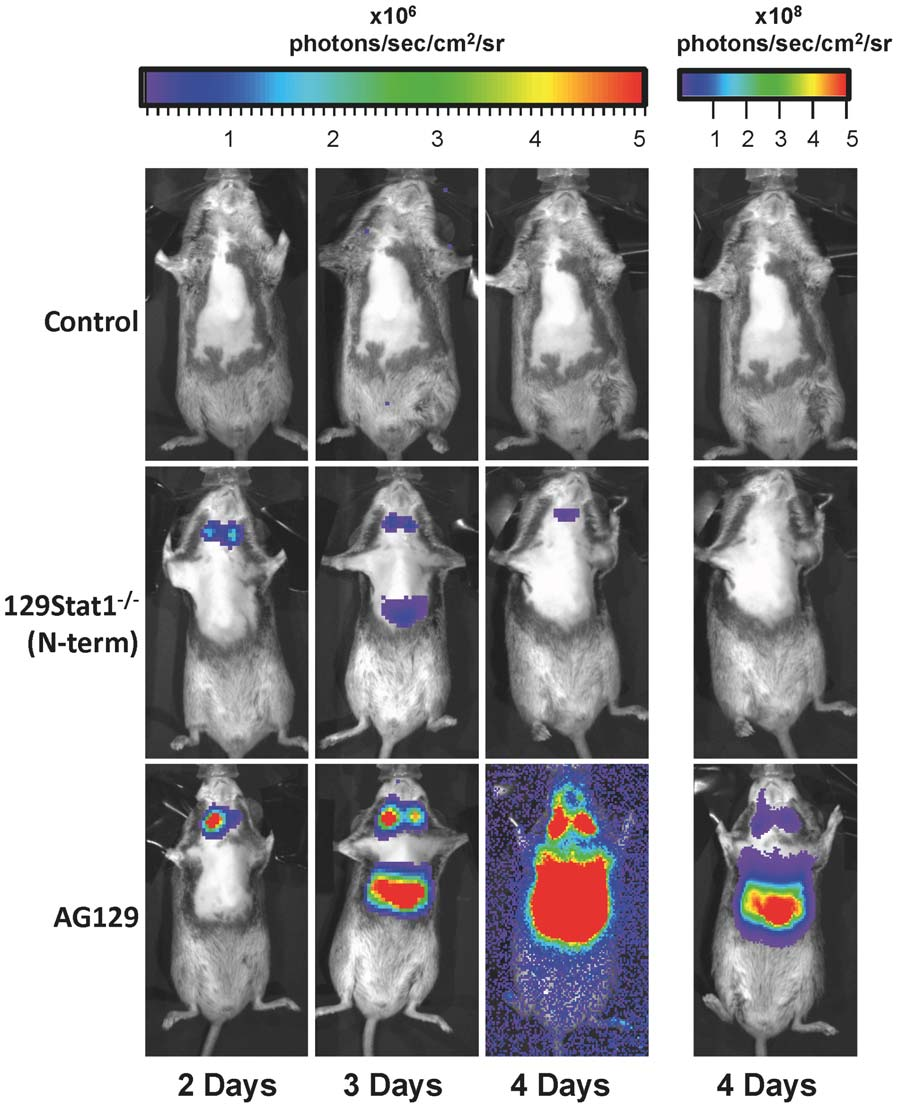
Pattern of viral spread in control, 129Stat1^−/−^(N-term), and AG129 mice assessed by bioluminescent imaging. *In vivo* bioluminescent imaging analysis was performed at 2, 3, and 4 dpi following corneal inoculation of 2×10^6^ pfu/eye of HSV-1 KOS/Dlux/OriL. Images are representative of 4 independent experiments. Daily images from the same mice per strain formatted on an identical photon flux scale are shown. For clarity additional images in the right hand column are shown for 4 dpi on a higher photon flux scale to avoid the saturated image for the AG129 mice.

Our results indicated the possibility that distinct mechanisms or tropisms were driving the disease and lethality in the virus-infected 129Stat1^−/−^(N-term) and AG129 mice. HSV-1 is considered a neurotropic virus, yet previous studies with AG129 mice revealed a disseminated infection that involved visceral organs [25], [30]. Dissemination of a related herpesvirus, pseudorabies virus, had also been shown in AG129 mice following intraperitoneal infection [34]. We used bioluminescent imaging to examine HSV-1 spread and tropism in 129Stat1^−/−^(N-term) mice using a luciferase reporter virus KOS/Dlux/OriL [35] (Fig. 2). Luciferase signal from the ocular region was present in all mice in accordance with previous reports of viral replication in the cornea [24], [27](data not shown).

While the control mice showed minimal luciferase activity, 129Stat1^−/−^(N-term) mice showed transient luciferase activity by 2 dpi in the draining cervical lymph nodes, and by 3 dpi in the liver and spleen. As reported previously, the AG129 mice infected with the KOS/Dlux/OriL virus exhibited a disseminated infection [25]. Unlike the 129Stat1^−/−^(N-term) mice, the AG129 mice did not clear the infection of the draining cervical lymph nodes, liver, or spleen. These organs showed high levels of luciferase activity that were sustained until the death of the animal on 5 dpi.

When the 129Stat1^−/−^(N-term) tissues were examined by bioluminescent imaging *ex vivo*, a transient signal was localized to the liver and spleen on 3 dpi, and signals were evident in the trigeminal ganglia and brain stem on 3 and 6 dpi (Fig. S1). Positive signals were also found in the brain, brain stem, trigeminal ganglia, liver, and spleen of infected AG129 mouse tissues at 3 dpi (Fig. S1). In addition, luciferase signal was low or undetectable in intestines, stomach, and surrounding gut tissues (data not shown). Two regions, the neck and the abdomen, were identified during *in vivo* imaging and selected for quantification of the bioluminescent signal. The signal in the neck was determined by *ex vivo* imaging to be largely from the draining lymph nodes. The abdominal signal was derived from a combination of activity in the liver and spleen. In both regions of interest (ROI) analyses, the luciferase signal was markedly higher in the AG129 mice than in the control or 129Stat1^−/−^(N-term) mice (Fig. S2). Not unexpectedly, the lack of Stat1 or IFN receptors had a marked effect on control of disseminated infection. However, these results were notable for the differences seen between the ability of the deficient mice to control the disseminated, visceral infection. The bioluminescent imaging also supported our finding in the lethality analysis, that viral pathogenesis differs markedly in strain 129Stat1^−/−^(N-term) and AG129 mice.

### 129Stat1^−/−^(N-term) mice control hematogenous infection, but succumb to CNS infection

**Figure 3:**
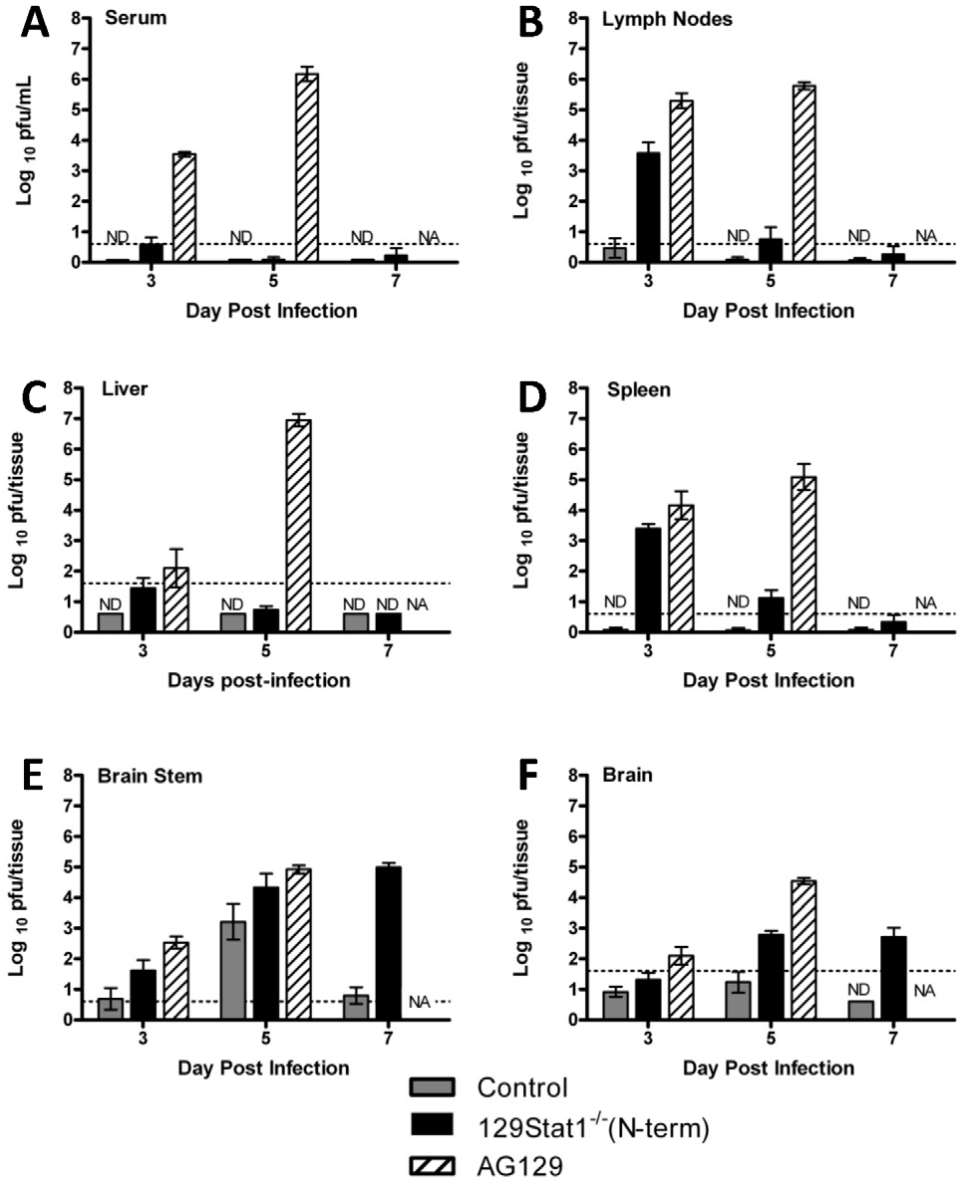
Pattern of viral spread in control, 129Stat1^−/−^(N-term), and AG129 mice assessed by viral titer in relevant tissues. Control, 129Stat1^−/−^(N-term), and AG129 infected via the cornea with 2×10^6^ pfu/eye of HSV-1 KOS were sacrificed at 3, 5, or 7 dpi for a viral titer analysis of tissues identified in the bioluminescent imaging. Each bar represents the tissue titer of at least 8 tissues from a minimum of 3 independent experiments. Titers are reported as pfu/ml (serum) or pfu/tissue (lymph nodes, liver, spleen, brain stem and and brain. The dashed line indicates limit of detection. ND = Not detected. NA = mouse not available.

To expand and confirm both previous studies and the bioluminescent imaging results, viral titers were measured in serum, draining lymph nodes, liver, spleen, and CNS tissues [24], [27]. In control mice, low titers of virus were found in the lymph nodes at 3 and 5 dpi (Fig. 3B), while virus was undetectable in the liver, spleen, and serum (Fig. 3A, C and D). The data showed a transient visceral infection in 129Stat1^−/−^(N-term) mice that peaked at 3 dpi in the lymph nodes, liver, spleen, and serum, correlative with the bioluminescent imaging data. In contrast, the AG129 mice showed detectable viral replication in the lymph nodes as early as 1 dpi (data not shown), and viral titers in the liver, spleen, and serum increased dramatically in the five days following infection, with no evidence of control or clearance (Fig. 3A, C and D).

Viral titer analyses of CNS tissues revealed an interesting different pattern of infection (Fig. 3). Control mice cleared the virus from the CNS by 7 dpi. In contrast to visceral tissues, 129Stat1^−/−^(N-term) mice could not clear virus from their CNS (Fig. 3 and [26]. Instead, viral replication increased in the brain and brain stem out to day 5, and then maintained a constant level out to time of death, consistent with the *ex vivo* bioluminescent imaging analysis (Fig. S1). Additionally, these mice showed consistently increased blood-brain barrier permeability by Evans blue dye uptake, consistent with CNS inflammation and damage (data not shown). CNS titers from the tissues of infected AG129 also showed increasing viral titers over time and a lack of viral clearance, although there were no changes in blood-brain barrier permeability on day 4, just prior to death (data not shown). These results indicated that the efficacy of the IFN-dependent antiviral response differed between the visceral and CNS tissues.

### Analysis of infected cells in lymphoid organs of AG129 mice

**Figure 4:**
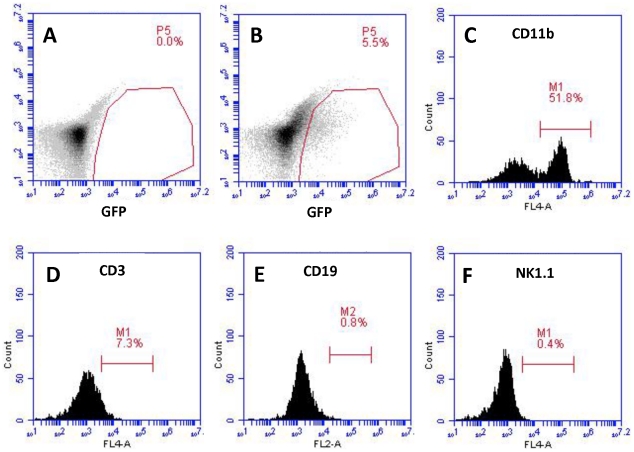
Flow cytometry. Identification of the infected cellular subsets within the spleens of AG129 mice. Single-cell suspensions of spleens from uninfected (A) and AG129 mice infected with KOS64gfp for 96 hours (B) were evaluated for virally-expressed gfp fluorescence by FACS analysis. To discern which cellular subsets were infected, the gfp^+^ cells gated in (B) were analyzed for (C) CD11b, (D) CD3, (E) CD19 and (F) NK1.1 expression. Similar results were observed in multiple mice between 96–100 hours post-infection.

When AG129 mice were infected via the cornea with KOS64gfp, >5% of their splenocytes were gfp-positive by flow cytometry at 4 days postinfection (compare Fig. 4A to 4B). In contrast, gfp-positive splenocytes were undetectable by flow cytometry in control and 129Stat1^−/−^(DBD) mice (data not shown). Titering of serum and of separated cells and supernatants from infected spleens, revealed high levels of virus in the serum of infected mice (Fig. 3A), and that there was 5- to 10-fold more virus in the cell-free fraction of infected spleens than in the cell-associated fraction (data not shown). The gfp-positive splenocytes from the AG129 mice were subsequently analyzed by flow cytometry to identify the infected cellular populations. CD11b^+^ cells were the predominant cellular subset infected (>62% of gfp^+^ cells were CD11b^+^; Fig. 4C). In contrast, few if any T cells (identified by CD3; Fig. 4D), B cells (CD19; Fig. 4E) or NK cells (NK1.1; Fig. 4F) were gfp-positive. These data identify the CD11b^+^ cells, likely splenic macrophages and potentially neutrophils, as both the principal and selective reservoir of infection in the spleen.

### 129Stat1^−/−^(N-term) bone marrow dendritic cells (BMDCs) respond to exogenous Type I IFN *in vitro*

**Figure 5:**
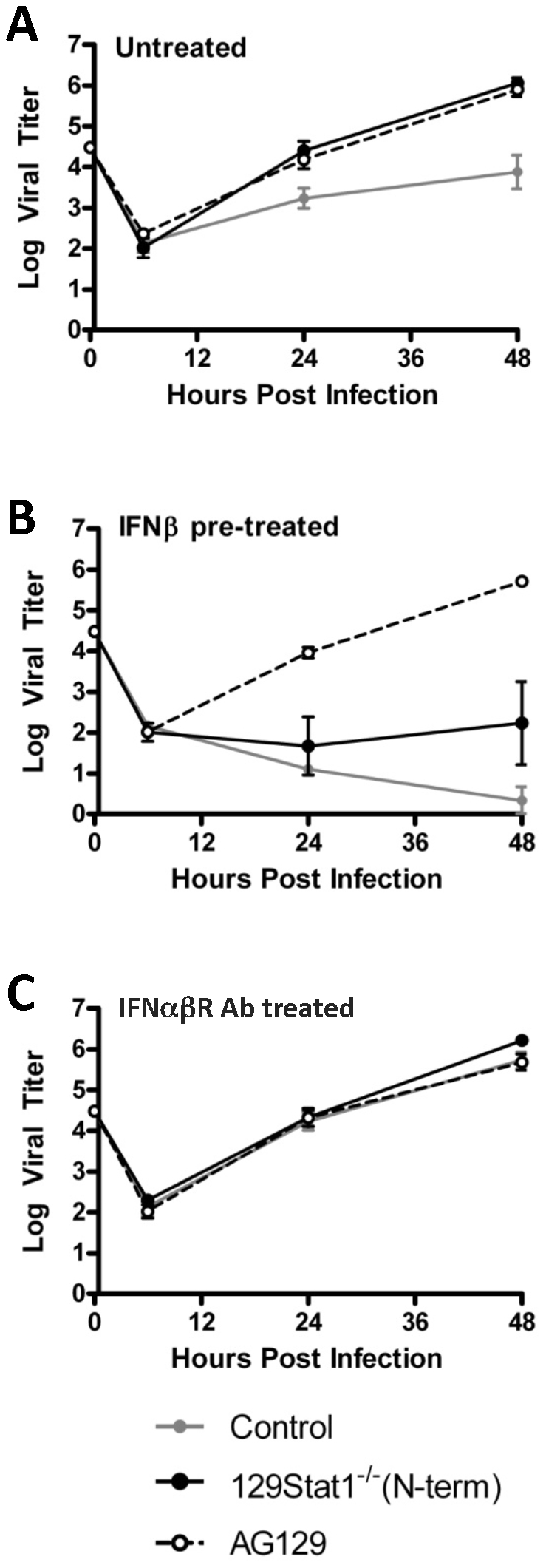
BMDCs from 129Stat1^−/−^(N-term) mice respond to exogenous Type I IFN. In vitro BMDC cultures were left untreated (A), pre-treated with 1000 U/mL of IFN-ß (B), or pre- and post-treated with 5 ug/mL of the IFNAR blocking antibody MAR1-5A3 (C). Cells were infected at an MOI of 0.01 with HSV-1 KOS, and cells and supernatents were harvested at 6, 24, and 48 hpi for a viral titer analysis. Each point represents a minimum of 3 measurements from at least 3 independent experiments.

As shown above, the strain 129Stat1^−/−^(N-term) mice exhibited control of a visceral, but not CNS infection, indicating that either activation or efficacy of an antiviral pathway might be occurring in a tissue-specific fashion. To characterize which pathway might be utilized, we examined the ability of isolated BMDCs from the deficient mice to control an HSV-1 infection *in vitro*. Stat1-deficient BMDCs have been shown to support increased HSV-1 replication relative to wild-type control cells [36]. Furthermore, AG129 BMDCs are more susceptible than 129Stat1^−/−^(N-term) BMDCs to Dengue virus and Sendai virus, and 129Stat1^−/−^(N-term) BMDCs produce IFN-α and IFN-γ in response to infection [22]. Consistent with a previous report [37], untreated BMDC's derived from 129Stat1^−/−^(N-term) and AG129 mice were similarly more productive for HSV-1 infection in a multi-step growth curve analysis, with titers in the deficient cells nearly 100-fold greater than titers in cells derived from control mice (Fig. 5A). Pre-treatment of BMDC's with exogenous IFN-ß rapidly suppressed viral replication in control cells but had no effect on replication in AG129 cells (Fig. 5B). Surprisingly, pre-treatment of 129Stat1^−/−^(N-term) BMDCs with exogenous IFN-ß reduced viral replication by approximately 1000-fold, demonstrating a strong antiviral response in these cells. Similarly, a 100–1,000 fold reduction of HSV and VSV titers were observed in IFN-ß pretreated MEFs derived from 129Stat1^−/−^(N-term) mice (data not shown). Pre-treatment of BMDC's with a Type I IFN receptor blocking antibody [38] restored viral replication in all three cell types to that of the untreated 129Stat1^−/−^(N-term) and AG129 levels, indicating that the production of IFN in the control cell cultures was a key factor in controlling HSV replication in these cells (Fig. 5C). This analysis therefore supports the hypothesis that the 129Stat1^−/−^(N-term) BMDCs respond to Type I IFN pre-treatment, and suggested that this response was dependent on the Type I IFN receptor.

### Bioluminescent imaging of 129Stat1^−/−^(N-term) mice treated with IFNαß receptor (IFNAR) blocking antibody

The above results indicated that 129Stat1^−/−^(N-term) BMDCs respond to exogenous Type I IFN via the IFNAR. This is consistent with the idea that an IFNAR-dependent response is the mechanism by which the strain 129Stat1^−/−^(N-term) mice clear the disseminated infection. To test this possibility, control and 129Stat1^−/−^(N-term) mice were pre-treated i.p. with an IFNAR blocking antibody. Mice were then infected via the corneal route with KOS/Dlux/OriL virus, and monitored by bioluminescent imaging.

**Figure 6:**
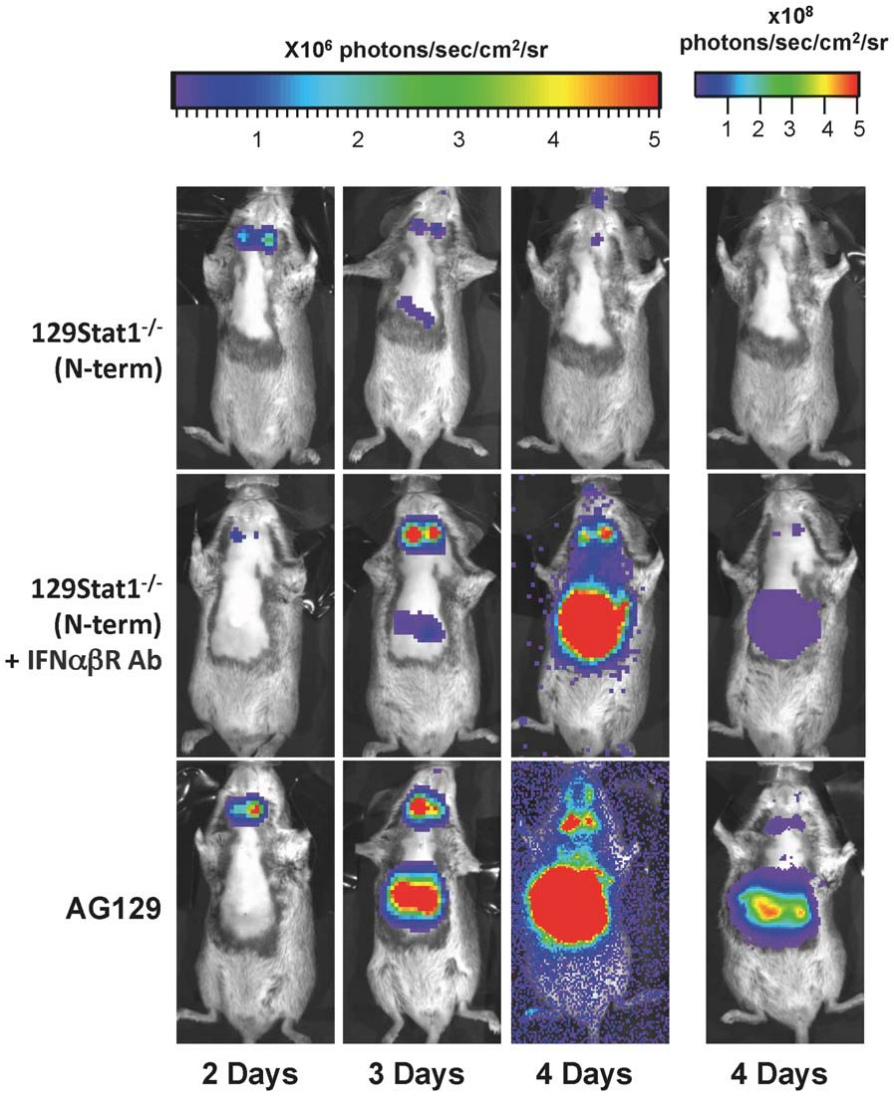
129Stat1^−/−^(N-term) mice treated with anti-Type I IFN receptor antibody display visceral infection similar to AG129 mice. *In vivo* bioluminescent imaging analysis of untreated 129Stat1^−/−^(N-term), IFNαß receptor blocking antibody (MAR1-5A3) treated 129Stat1^−/−^(N-term), and untreated AG129 mice infected with 2×10^6^ pfu/eye of HSV-1 KOS/Dlux/OriL. Bioluminescent imaging analysis was performed as described above. A minimum of 5 mice were analyzed and representative images shown. Daily images from the same mice per strain formatted on an identical scale are shown. For clarity, additional images in the right hand column are shown for 4dpi on a higher photon flux scale to avoid the saturated image for the AG129 mice.

IFNAR antibody-treated control mice showed increased bioluminescent imaging signals, with bioluminescent signal visible in the draining lymph nodes between 2 and 4 dpi (data not shown). Light intensity, however, was still less than that observed in AG129 mice. In contrast, IFNAR antibody-treated 129Stat1^−/−^(N-term) mice showed up to a 1,000 fold increase in photon flux, and closely resembled the AG129 in terms of their inability to clear the disseminated visceral infection (Fig. 6). An ROI analysis of the lymph node and abdominal region of these mice showed levels of bioluminescent signal comparable to those found in the AG129 mice (Data not shown). Furthermore, the antibody-treated 129Stat1^−/−^(N-term) mice died by 5 dpi, identical in time to that seen in AG129 mice. These data demonstrate that the strain 129Stat1^−/−^(N-term) mice had residual signaling via the IFNAR that was sufficient to alter both viral tropism and survival following HSV-1 infection.

### Bioluminescent imaging of 129Stat1^−/−^(N-term) and 129Stat1^−/−^(DBD) mice

The results so far in this study indicated a residual ability in the strain 129Stat1**^−/−^**(N-term) mice to respond to Type I IFN via the IFNAR. Possible explanations for these observations include IFNAR-dependent, Stat1-independent redundant pathway for Type I IFN signaling, or possibly residual Stat1-dependent activity. To address this, first, the 129Stat1^−/−^(N-term) mice were genotyped to eliminate the possibility that errors in animal husbandry had caused generation of a heterozygous, or reverted mouse line (data not shown). Next, we infected a second Stat1**^−/−^** mouse, 129Stat1^−/−^(DBD) (kindly provided by Joan Durbin), which contains a deletion in the Stat1 DNA binding domain [13]. Similar to the 129Stat1^−/−^(N-term) strain this Stat1-deficient mouse strain also has increased susceptibility to pathogens, and a defective IFN response [13], [22], [32], [39].

**Figure 7:**
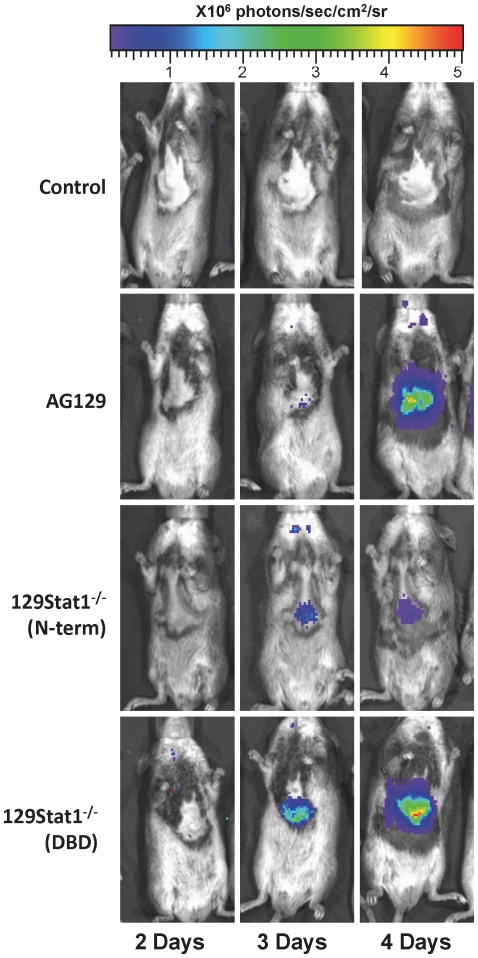
Bioluminescent imaging comparison of 129Stat1^−/−^(N-term) and 129Stat1^−/−^(DBD) mice. *In vivo* bioluminescent imaging analysis of control, AG129, 129Stat1^−/−^(N-term) and 129Stat1^−/−^(DBD) mice was performed on days 2, 3 and 4 postinfection. Daily images from the same mice per strain formatted on an identical scale are shown. Images shown are representative of three independent experiment with at least 2 mice per group per experiment.

**Figure 8:**
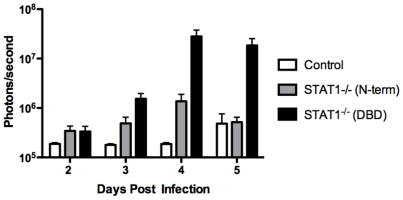
Region of interest (ROI) analysis of abdominal bioluminescence. Analysis of control, 129Stat1^−/−^(N-term) and 129Stat1^−/−^(DBD) mice (open, gray, and black bars respectively) from 3 independent experiments**.** Identical boxes surrounding the tissues of interest (spleen and liver) were drawn with Living Image and IgorPro software, and bioluminescent signal reported in photons/sec.

To compare the Stat1^−/−^ mouse lines, strain 129Stat1^−/−^(N-term) [12] and 129Stat1**^−/−^**(DBD) [13] mice were infected with KOS/Dlux/OriL as described above and examined daily by bioluminescent imaging (Fig. 7). In both mouse strains, an infection was detected in the draining lymph nodes by 2 dpi and in the liver and spleen by 3 dpi. As seen previously, by 4 dpi the generalized infection was controlled in the 129Stat1**^−/−^**(N-term) mice. In contrast, the 129Stat1**^−/−^**(DBD) mice failed to control the generalized infection. The pathogenesis of HSV-1 in the 129Stat1**^−/−^**(DBD) mice was similar to that seen in AG129 mice and to that seen in strain 129Stat1**^−/−^**(N-term) mice treated with the IFNAR blocking antibody (Fig. 6). ROI analysis of the bioluminescent signals revealed 10–50 times higher photon flux in the 129Stat1**^−/−^**(DBD) than the 129Stat1^−/−^(N-term) mice (Fig. 8). Moreover, almost 100-fold more virus (5×10^5^ pfu/ml of homogenate compared with 7×10^3^ pfu/ml) was found in the livers of 129Stat1^−/−^(DBD) mice than in 129Stat1^−/−^(N-term) (data not shown). To eliminate the caveat that the differences in HSV pathogenesis observed in these two versions of Stat1-deficient mice were due to differences in background between the two strains, we performed SNP analysis (http://www.dartmouse.org/). 129Stat1^−/−^(DBD) mice showed greater than 97% SNP identity to 129 Sv/Ev control mice, while the 129Stat1^−/−^(N-term) mice had greater than 99% identity to strain 129 (data not shown). As an additional control, we also tested the 129Stat1^−/−^(DBD) mouse line in the C57Bl6 background and showed increased bioluminescent imaging and titers relative to 129Stat1^−/−^(N-term) (Figure S2, and data not shown). Together these data eliminate the possibility that the differences between 129Stat1^−/−^(N-term) and 129Stat1^−/−^(DBD) are due to subtle background differences in the two 129 knockout strains [40], [41].

## Discussion

The role of the interferon response in controlling HSV infection is well established, but precise roles for certain IFN pathway components in the determination of tropism have not been determined. In humans, herpetic encephalitis is a frequently observed viral CNS infection, while herpetic hepatitis in humans is relatively rare, accounting for <2% of viral-induced acute liver failure. It is most often observed in immunocompromized adults, in neonates, or during the final trimester of pregnancy. Diagnosis of HSV in such cases is often missed and effective antiviral treatment is therefore delayed, thus resulting in a high (∼74%) fatality rate [8]. The utility, therefore, of the AG129 mouse as a model for herpetic hepatitis is now under further investigation.

Stat1^−/−^ mice have been utilized in a large number of studies to delineate the roles of Stat1 and IFN in controlling infection and mediating IFN-dependent gene expression. Many studies have shown increased replication in Stat1^−/−^ cells and mice infected with a variety of pathogens in including *Listeria monocytogenes*, vesicular stomatitis virus, herpes simplex virus, Dengue virus, Sendai virus, mouse norovirus, Sindbis virus, *Leishmania major*, yellow fever virus, and mouse cytomegalovirus [12], [18], [19], [20], [21], [22], [23], [26], [27], [33], [42], [43]. Overall, these studies showed a similar pattern of increased replication in target tissues from a variety of inoculation routes. Several of these studies also reported IFN-dependent gene expression patterns that were categorized as Stat1-independent pathways. A recent study indicates that much of this Stat1-independent activity is dependent on Stat2, since mice lacking both Stat1 and Stat2 show susceptibility to dengue virus that is equivalent to AG129 mice [31]. It is unclear, however, from all of these studies whether the IFN-dependent gene expression wholly arose from Stat1-independent pathways, or whether it might have resulted, in part, from a small amount of residual Stat1 activity, especially in the 129Stat1^−/−^(N-term) mice. Despite this possibility, 129Stat1^−/−^(N-term) cells lack the expected responses to IFN treatment with changed expression of IFN-dependent genes, decreased virus-induced apoptosis, altered IFN-dependent upregulation of cell surface markers, and increased susceptibility to pathogens [23], [44], [45], [46]. It is clear therefore, that the residual activity is only relevant or detectable under certain specific infection conditions. Moreover, these mice succumb to HSV encephalitis, a tropism that closely resembles that seen in some Stat-deficient humans [4]. These mice represent a closer model of generalized human HSV infection than the 129Stat1^−/−^ (DBD) which usually succumb to hepatitis. These mice remain, therefore, a tractable and useful model of innate immune insufficiency. Our studies, and studies of several other labs, have consistently shown greater susceptibility to pathogens in the AG129 mice as compared to the 129Stat1^−/−^(N-term) mice [18], [22], [23], [30], [33]. Indeed, the profound viscerotropism of a variety of viruses in AG129 mice leads to the general conclusion that Type I and Type II IFN receptors are essential for protection of visceral organs from viral infection. It is possible, based on our flow cytometry data, that this viscerotropism may result from increased permissivity of monocytes and macrophages which are highly infected in these mice. These immune cells may serve as a conduit in which the virus can replicate and spread, and thereby traffic to, and replicate in the lymph nodes, spleen, liver and other visceral organs. Previous studies have demonstrated that HSV can replicate to low levels in leukocytes, [47], [48], [49], [50] and this study suggests that these cells become more permissive and productive in the absence of IFN responses. This is also consistent with a previous study of Dengue virus in AG129 mice in which increased virus replication was demonstrated in CD11b+ (and other) cells in the spleen [51].

The Stat1-deficient strains examined in this study have distinct Stat1-deficient alleles. The 129Stat1^−/−^(N-term) mouse lacks the N-terminal protein-protein interaction domain [12], whereas the 129Stat1^−/−^(DBD) [32] and B6Stat1^−/−^(DBD) [13] are obviously identical, lacking the DNA binding domain of Stat1 [13]. Few direct comparisons of these mice have been reported, but Shresta *et al* infected 129Stat1^−/−^(N-term) and 129Stat1^−/−^(DBD) mice with Dengue and showed that *both* strains were resistant to infection relative to AG129 mice [22]. They also found production of IFN-α in BMDC cultures following infection of 129Stat1^−/−^(N-term) cells with Dengue and Sindbis virus and from their results they concluded that Stat1-independent pathways were mediating IFN expression in these cells. They did not, however, perform the IFN-α production experiments on the 129Stat1^−/−^(DBD) mice. Experiments in this study indicate that both the 129Stat1^−/−^(N-term) and 129Stat1^−/−^(DBD) mice produce IFNα in response to virus infection (data not shown) suggesting that it is the response to IFN that differs between these mouse strains, rather than IFN production itself. The 129Stat1^−/−^(N-term) mice have an N-terminal deletion in Stat1 that reduces the molecular weight from 91 kDa to 72 kDa, diminishes expression of the truncated protein to 4–7% of the native Stat1 protein, and reduces transcript levels to 4% of normal levels [12]. It was suggested that an untranslated exon 3kb upstream of the Stat1 AUG could result in expression of a mutant form of Stat1 in the gene targeted mice that initiates at position 135. This protein under most circumstances is expressed in very limited quantities, and is, for the most part, functionally inactive. In response to IFN-γ treatment, the truncated Stat1 protein bound DNA at <2% of wild-type Stat1 levels. Based on experiments with IFN-α and IFN-γtreatment, the originators of this mouse concluded that these mice produced a limited amount of a mutated Stat1 protein that was functionally inactive [12]. The results in this current study suggest, however, that what might be taken as Stat1-independent, IFN-dependent signaling may in fact be partly due to residual Stat1 signaling via the Type I IFN receptor. This hypothesis is best supported by the observation that antiviral activity is abrogated in the presence of the Type I IFN receptor-blocking antibody. Additional supporting data include the observation that the 129Stat1^−/−^(DBD) and B6Stat1^−/−^(DBD) are significantly more susceptible to HSV infection than the 129Stat1^−/−^(N-term) mice. It is likely, therefore, that the Stat1^−/−^(DBD) mice represent a more complete knockout of Stat1 function than the Stat1^−/−^(N-term) mice. An alternate, non-mutually exclusive hypothesis, is based on the fact that HSV inhibits Stat1 activity, and that it is more capable of inhibiting the residual antiviral activity of the Stat1^−/−^(DBD) mice than the Stat1^−/−^(N-term) mice [52], [53], [54], Further characterization of possible residual Stat1 signaling for both Type I and Type II IFN in the strain 129Stat1^−/−^(N-term) mice will be critical for interpretation of all results based on these mice because, as shown here, this Stat1 activity is sufficient to significantly alter HSV-1 pathogenesis. Whether the relative resistance of 129Stat1^−/−^(N-term) mice to visceral infection is due to Stat1-independent pathways, or residual Stat1 activity, is a subject for further study.

This study also builds on earlier work with mice lacking the Type I IFN receptor (IFNAR^−/−^ or A129 mice) or the Type II IFN receptor (IFNGR^−/−^ or G129 mice) [25]. Previously, A129 mice infected with HSV-1 via the cornea successfully controlled a transient visceral infection with 100% survival. G129 mice fully controlled HSV-1 infection, while AG129 showed no control over the virus [25]. From this it was concluded that the Type II IFN response alone played a minor role in controlling acute HSV-1 infection, but played a synergistic role with Type I IFN as shown by the susceptibility of the AG129 mice. A particularly interesting finding from the current study was that antibody blockade of the IFNAR in the 129Stat1^−/−^(N-term) mouse was sufficient to shift viral tropism to the liver. This strongly supports the idea of a role for Type I IFN in controlling HSV-1 infection in the viscera. We propose that the IFNAR antibody-treated 129Stat1^−/−^(N-term) mice have a completely abrogated Type I IFN function, while Type II IFN function is only partially reduced due to the incomplete knockout of Stat1 function in these mice. From these data, we can assemble a hierarchy of the mouse models in terms of their ability to control an HSV-1 infection: Control 129 mice > G129> A129 >129Stat1^−/−^(N-term) >129Stat1^−/−^(DBD)  =  B6Stat1^−/−^(DBD)  =  129Stat1^−/−^(N-term) with IFNAR antibody  =  AG129.

Finally, our data underscore the utility of bioluminescent imaging as a rapid screen for changes in viral tropism in mouse models, and provide us with a tractable model with which to study the impact of critical components of the innate immune system in the protection of the brain and liver from herpetic infection. In addition, our data show that normally benign viruses can be very dangerous in immune-deficient hosts, and that the ability to clear such viruses is dependent upon an intact innate response. The relative contribution of direct viral damage, as opposed to immune-mediated damage in these model systems is the subject for future studies, and has important implications for vaccine design, and prevention of immunopathologic disease.

## Materials and Methods

### Cells, viruses, and mice

Vero cells were used for amplification and titering of viral stocks as previously described [55]. The viruses used in this study were in the KOS background [56]. Construction of the HSV-1 KOS/Dlux/oriL luciferase-expressing virus was previously described [57]. KOS64gfp (VanHeyningen and Leib, unpublished data) contains a cassette comprising the HCMV promoter driving gfp inserted into the *Bgl* II site at the map position previously described for KOS6B [58]. Mock-treated animals were inoculated with uninfected Vero cell lysates prepared in a parallel manner as infected Vero lysates. Mouse strains used included the control 129S6 as wild type mice (Taconic Farms, Germantown, NY), 129 S6 Stat1 knock-out mice lacking the N-terminal domain (129Stat1^−/−^(N-term)) [12], 129 IFNαßγR^−/−^ mice (AG129) [15]. These mice were compared to Stat1 deficient mice lacking the DNA binding domain in both 129, referred to as 129Stat1^−/−^(DBD) (kindly provided by Joan Durbin) [32], and C57BL/6 (B6Stat1^−/−^(DBD)) backgrounds [13]. 129Stat1^−/−^(N-term) mice were genotyped to ensure proper husbandry. The genetic backgrounds of the 129Stat1^−/−^(DBD) mice were additionally assessed at the DartMouse™ Speed Congenic Core Facility at Dartmouth Medical School. DartMouse uses the Illumina, Inc. (San Diego, CA) GoldenGate Genotyping Assay to interrogate 1449 SNPs spread throughout the genome. The raw SNP data were analyzed using DartMouse's proprietary SNaP-Map™ and Map-Synth™ software, allowing the determination for each mouse of the genetic background at each SNP location. Mice were housed in the Washington University School of Medicine and Dartmouth Medical School barrier facilities, and infected in the respective biohazard facilities. At both facilities, sentinel mice were screened every 3 months and determined to be negative for adventitious mouse pathogens, in particular mouse norovirus. Mice were infected between 6–8 weeks of age. Mice were housed, treated, and euthanized when necessary in accordance with all Federal and University policies.

### Animal infection procedures

For corneal infection, equivalent numbers of 6–8 week old male and female mice were anesthetized intraperitoneally with ketamine (87 mg/kg body weight) and xylazine (13 mg/kg). Corneas were bilaterally scarified with a 25G syringe needle and virus was inoculated by adding 2×10^6^ pfu in a 5 µl volume [55]. Mice were sacrificed at the specified time post-infection for determination of tissue viral titers or sample harvest. Eye swab material was collected for a standard plaque assay as previously described [55]. Liver, spleen, cervical lymph nodes, brain, brain stem, and serum were collected. All tissues were harvested into an appropriate volume of media and stored at −80°C. Tissue homogenization was accomplished with 1 mm or 3 mm glass beads. Whole blood was collected from infected mice at the indicated time post-infection. Serum was isolated using BD Microtainer serum separator tubes (Becton Dickinson, Franklin Lakes, NJ) and viral load in serum was determined by plaque analysis on Vero cells. Titers were derived from at least eight animals and collected from three or four independent groups of mice. Tissue titers are reported as pfu per tissue, except for the cervical lymph nodes, in which two tissues were combined into one sample. Selected animals were pre-treated with 2.0 mg i.p. of anti-mouse IFN α/ß receptor (Clone MAR1-5A3; IFNAR1 subunit) antibody (Leinco Technologies Inc, St Louis, MO) one day prior to corneal infection, and re-treated again with 0.2 mg antibody at day 3 post-infection [38].

### Bioluminescence imaging

Mice infected via the corneal route with KOS/Dlux/OriL were imaged with a cooled charge-coupled device (CCD) camera-based bioluminescence (IVIS 100, Caliper LifeSciences, Hopkinton, MA) as previously described [35]. For bioluminescence imaging of living animals, infected mice were injected intraperitoneally with 150 µg/g D-luciferin (Biosynth, Naperville, IL) in PBS, anesthetized with 2.5% isofluorane, and imaged (exposure time 1–60 seconds, binning 8, field of view 15 or 19.6, f/stop 1 or 2, open filter, anterior side). Signal was displayed as photons/sec/cm^2^/sr. For analysis, regions of interest (ROI) were defined manually around the lymph nodes, liver and spleen, and perinasal cavity and photon flux (photons/sec) calculated using Living Image and IgorPro Software (Version 2.50). Ex vivo images were obtained immediately after live imaging.

### Flow Cytometry

Mice were infected via the cornea with 2×10^6^ pfu per eye of KOS64gfp. Spleens were removed 100 hours postinfection and single-cell suspensions were generated by passage through a 70 µM cell strainer (BD Biosciences, San Jose, CA). The cellular fraction was treated with ACK lysis buffer (0.15 M NH4Cl, 1.0 mM KHCO3, 0.1 mM EDTA) to remove red blood cells, and the remaining cells were resuspended in 0.5% BSA in PBS or media for analysis. Cells were pre-incubated with Fc-blocking antibody (clone 2.4G2) prior to antibody staining. Anti-mouse antibodies against CD3 (145-2C11), CD11b (M1/70), NK1.1 (PK136), and CD19 (1D3) were purchased from eBioscience (San Diego, CA). Flow cytometry was done on the Accuri C6 and analyzed using CFlow and FlowJo 8.8.2 software.

### BMDCs

Bone marrow dendritic cells were harvested as previously described [37]. Briefly, bone marrow was flushed from both femurs of adult mice and cultured in RPMI supplemented with 10% FBS, Glutamax, sodium pyruvate, 250 U of penicillin/mL, 250 U of streptomycin/mL, and 2% GM-CSF. At 6 days post-harvest, BMDC's were collected, counted, and infected at an MOI = 0.01. BMDC's were pretreated 18 hrs prior to infection with recombinant mouse IFNß (PBL Interferon Source) at 1000 U/mL culture media or with anti-mouse IFN α/ß receptor (Clone MAR1-5A3; IFNAR1 subunit) antibody (Leinco Technologies Inc) at 5 µg/mL culture media.

## Supporting Information

Figure S1 ***Ex vivo*** **bioluminescence of infected tissues defines source of** ***in vivo*** **bioluminescence.** Immediately following *in vivo* bioluminescent imaging at 3 or 6 dpi, 129Stat1^−/−^(N-term) and AG129 infected with 2×10^6^ pfu/eye of HSV-1 KOS/Dlux/OriL were sacrificed and dissected to examine brain, brain stem, trigeminal ganglia, liver, and spleen using *ex vivo* bioluminescent imaging.
(TIF)

Figure S2 **Region of interest (ROI) analysis of lymph node and abdominal bioluminescence.** Images collected from control, 129Stat1^−/−^(N-term), AG129, 129Stat1^−/−^(N-term) treated with IFNαßR-blocking antibody, and B6Stat1^−/−^(DBD) mice were analyzed for bioluminescent signal by quantitation in two ROIs. Identical boxes surrounding the tissues of interest (cervical lymph nodes, spleen and liver) were drawn with Living Image and IgorPro software, and bioluminescent signal reported in photons/sec.
(TIF)
