# Supplementary figures and images for: Bioluminescent Imaging Reveals Divergent Viral Pathogenesis in Two Strains of Stat1-Deficient Mice, and in αßγ Interferon Receptor-Deficient Mice
Source: PLoS One. 2011 Sep 7;6(9):e24018. doi: 10.1371/journal.pone.0024018 (PMC3168466; doi:10.1371/journal.pone.0024018)

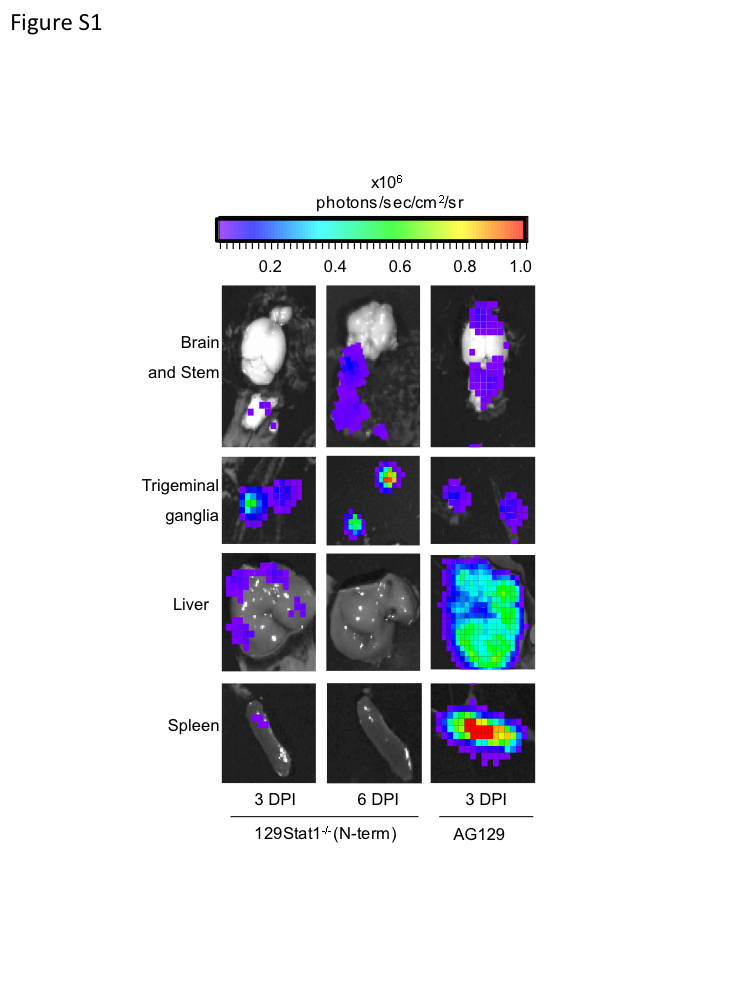

Supplement: Figure S1 — Ex vivo bioluminescence of infected tissues defines source of in vivo bioluminescence. Immediately following in vivo bioluminescent imaging at 3 or 6 dpi, 129Stat1−/−(N-term) and AG129 infected with 2×10^6^ pfu/eye of HSV-1 KOS/Dlux/OriL were sacrificed and dissected to examine brain, brain stem, trigeminal ganglia, liver, and spleen using ex vivo bioluminescent imaging. (TIF) [file pone.0024018.s001.tif]

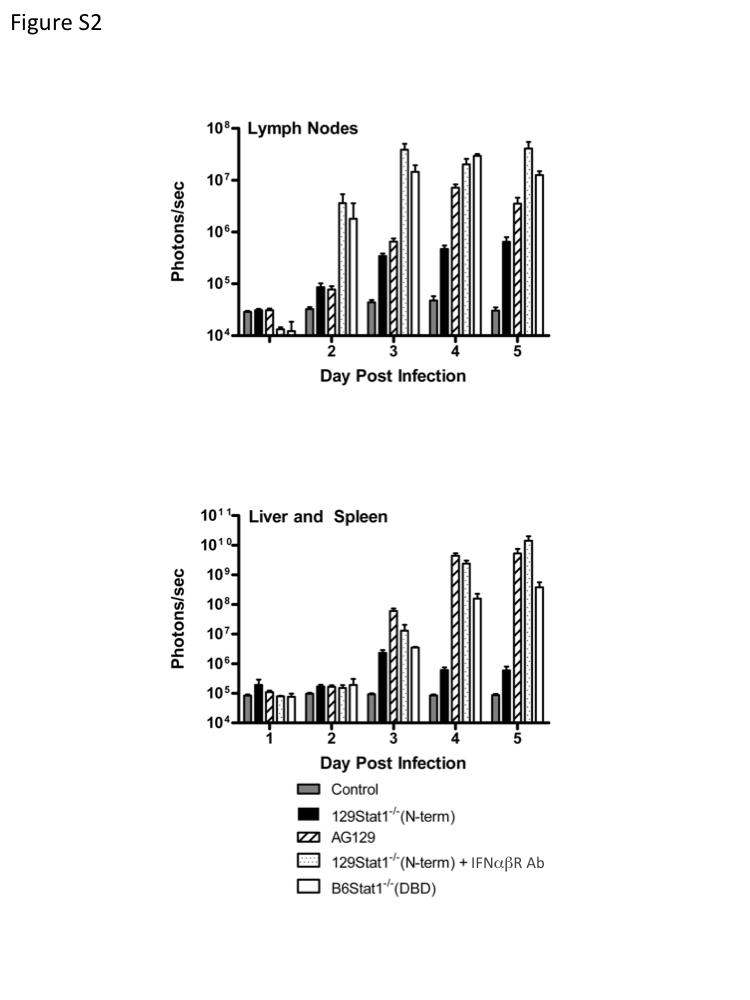

Supplement: Figure S2 — Region of interest (ROI) analysis of lymph node and abdominal bioluminescence. Images collected from control, 129Stat1−/−(N-term), AG129, 129Stat1−/−(N-term) treated with IFNαßR-blocking antibody, and B6Stat1−/−(DBD) mice were analyzed for bioluminescent signal by quantitation in two ROIs. Identical boxes surrounding the tissues of interest (cervical lymph nodes, spleen and liver) were drawn with Living Image and IgorPro software, and bioluminescent signal reported in photons/sec. (TIF) [file pone.0024018.s002.tif]
